# Foodborne infection of mice with *Salmonella* Typhimurium

**DOI:** 10.1371/journal.pone.0215190

**Published:** 2019-08-08

**Authors:** Olof R. Nilsson, Laszlo Kari, Olivia Steele-Mortimer

**Affiliations:** Laboratory of Bacteriology, Rocky Mountain Laboratories, National Institutes of Allergy and Infectious Diseases, National Institutes of Health, Hamilton, Montana, United States of America; University of Arkansas for Medical Sciences, UNITED STATES

## Abstract

The bacterial pathogen *Salmonella enterica* serovar Typhimurium is one of the most common causes of foodborne disease in humans and is also an important model system for bacterial pathogenesis. Oral inoculation of C57Bl/6 mice, which are genetically susceptible to *Salmonella*, results in systemic infection but the murine intestine is not efficiently colonized unless the intestinal microbiota is disrupted. Pretreatment of C57Bl/6 mice with streptomycin, followed by oral inoculation with *Salmonella* Typhimurium results in colitis resembling human intestinal *Salmonellosis*. The predominant method of delivery of bacteria is oral gavage, during which organisms are deposited directly into the stomach via a feeding needle. Although convenient, this method can be stressful for mice, and may lead to unwanted tracheal or systemic introduction of bacteria. Here, we developed a method for oral infection of mice by voluntary consumption of regular mouse chow inoculated with bacteria. Mice readily ate chow fragments containing up to 10^8^ CFU *Salmonella*, allowing for a wide range of infectious doses. In mice pretreated with streptomycin, infection with inoculated chow resulted in reproducible infections with doses as low as 10^3^ CFU. Mice not treated with streptomycin, as well as resistant Nramp1 reconstituted C57Bl/6J mice, were also readily infected using this method. In summary, voluntary consumption of chow inoculated with *Salmonella* represents a natural route of infection for foodborne salmonellosis and a viable alternative to oral gavage.

## Introduction

Bacteria belonging to the genus *Salmonella enterica* subsp. *enterica* are common causes of foodborne diarrheal disease [1] and a leading cause of death due to foodborne pathogens globally [2] and in the US [3]. Transmission occurs primarily via the fecal-oral route. *Salmonella enterica* serovar Typhimurium (hereafter *Salmonella*) is one of the serovars most commonly isolated from human gastrointestinal infections and is one of the most studied human bacterial pathogens. This, combined with its simple growth requirements, has led to its frequent use as a model organism for *in vivo* studies of the pathogenesis of gastrointestinal infections.

The most widely used animal model for *Salmonella* infection is the mouse [4]. Strains of mice differ in their susceptibility to *Salmonella*, with C57Bl/6J (B6) and BALB/c mice being highly susceptible and other strains, including 129/Sv, being very resistant [5–8]. Susceptibility is multifactorial, but one major resistance factor is the Nramp1 protein encoded by the *Slc11a1* gene [9]. Nramp1 is an ion transporter responsible for the transport of divalent cations out of phagosomes, thus limiting the availability of iron and other ions for ingested microbes and impairing their growth in phagocytes [10]. Many susceptible mouse strains, including B6, harbor a point mutation in the *Slc11a1* gene resulting in a non-functional Nramp1 protein [11, 12]. Oral infection of susceptible mouse strains with *Salmonella* leads to a systemic infection without efficient colonization of the intestine and the mice succumb within 5–6 days [13]. However, if the intestinal microbiota is disrupted by antibiotic treatment mice do develop intestinal inflammation more similar to human intestinal salmonellosis [14, 15]. Transgenic B6 mice expressing a fully functional allele of Nramp1 (B6N) are an alternative mouse model for host innate and adaptive responses to *Salmonella*. These mice develop a strong inflammatory response following oral infection and survive for several weeks [6, 16].

In the above models of oral *Salmonella* infection, the mice are almost always infected by oral gavage (OG), during which a blunt end gavage needle is used to deposit bacteria directly into the stomach. OG is widely used as a substitute for oral delivery since it allows for precise delivery of inoculum. However, there are drawbacks. Performing OG requires a moderate degree of technical expertise and can induce stress in mice, including raising corticosteroid levels in the blood or increasing blood pressure, which may affect study outcome [17–20]. Furthermore, mice may regurgitate delivered substances or infectious agents following gavage, resulting in tracheal or nasal administration [21, 22]. Lastly, gavage may induce pharyngeal or esophageal trauma, leading to the inadvertent delivery of substances or infectious agents directly into the blood stream as has been shown for *Listeria monocytogenes* [22–24].

Improvements to OG have been suggested, such as precoating needles with sucrose, which improved gavage success rate and reduced stress of animals [25]. Alternatively, a more natural method would be consumption of food or water containing a pathogen, which mimics the foodborne route of infection for *Salmonella* and would circumvent many of the drawbacks associated with OG. Indeed, when inoculated food was used to infect mice with *L*. *monocytogenes* systemic spread was delayed, compared to inoculation by OG, probably by avoiding direct systemic infection [24, 26]. However, to our knowledge, food as a vehicle of delivery for *Salmonella* infection has not been reported.

In this paper, we describe a foodborne infection method using regular mouse chow inoculated with *Salmonella*. This voluntary consumption (VC) mode of infection leads to consistent colonization in mice and eliminates many of the possible drawbacks associated with OG. Importantly, this method represents a natural route of infection with *Salmonella*.

## Results and discussion

### *Salmonella* survival on mouse chow

**Fig 1:**
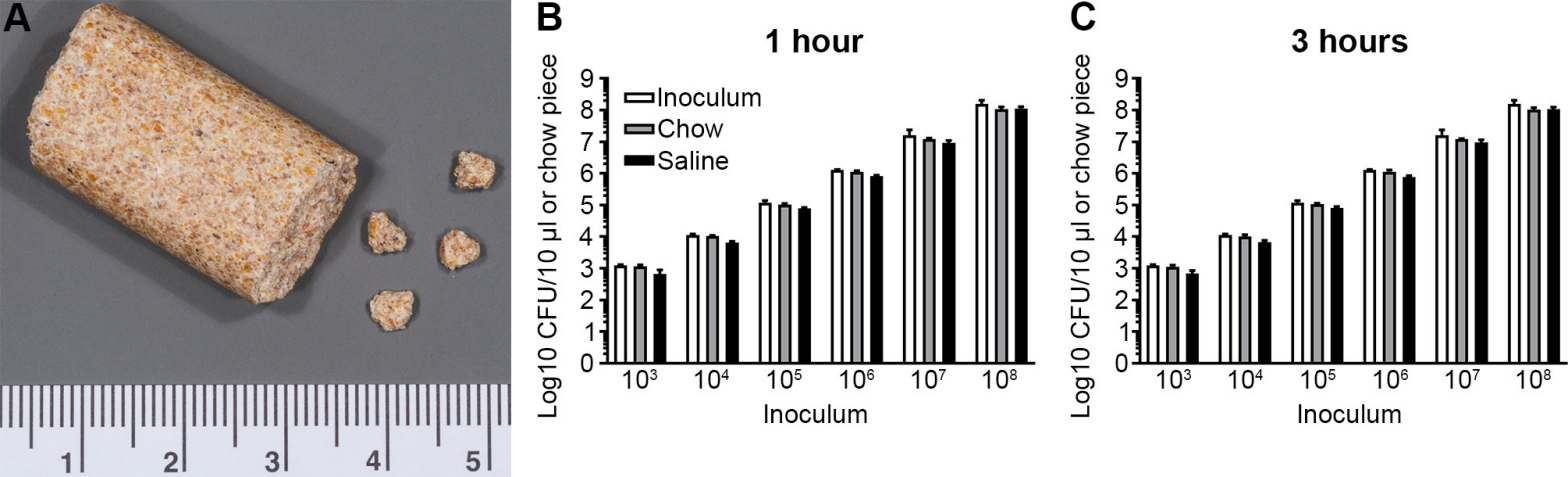
*Salmonella* survival on mouse chow. (A) Representative image of a regular mouse chow pellet and prepared chow fragments. Ruler scale in centimeters. (B, C) Survival of *Salmonella* on chow and in SPGS, 1 and 3 h after inoculation. Original inoculum added as a comparison. Data represent the mean ± SD of three independent experiments (n = 1 per experiment).

As a first step in testing whether mouse chow can be used for foodborne infection of mice, we tested the survival of *Salmonella* on chow. Fragments (30–45 mg each) of chow, prepared from pellets using a small hammer and forceps (Fig 1A), were inoculated with 10 μl of *Salmonella* suspended in sterile pharmaceutical grade saline (SPGS) and then incubated at room temperature for 1 or 3 h. The chow fragments were then homogenized, diluted and plated to enumerate colony forming units (CFUs). For comparison, bacteria were inoculated in SPGS alone. No decrease in viability of *Salmonella* was observed over the course of 3 h (Fig 1B and 1C).

### Comparison of OG and VC inoculation methods

**Fig 2:**
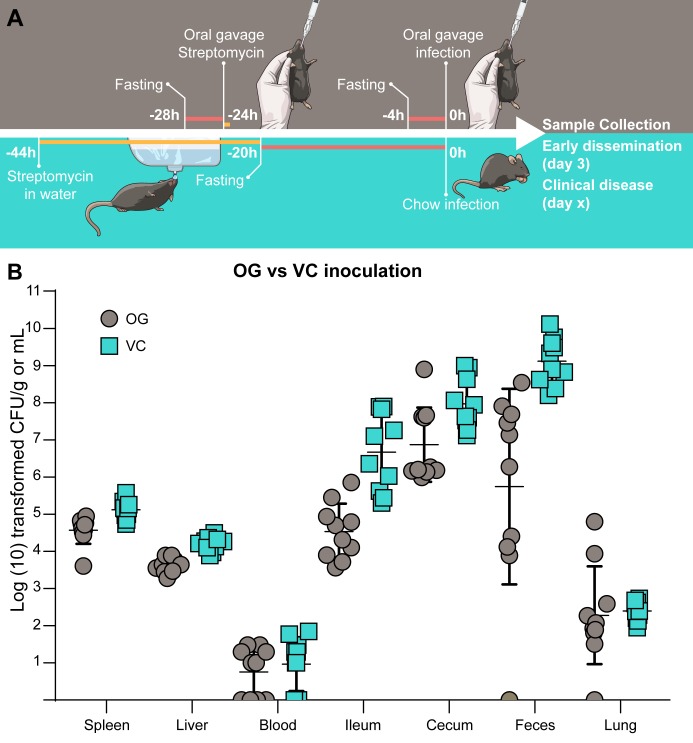
Comparison of tissue loads in mice infected by VC and OG. (A) Schematic representation of the OG (top) and VC (bottom) infections. (B) Bacterial numbers in tissues from mice infected either by OG or VC 3 days p.i. n = 10 mice. Symbols represent individual mice. Error bars represent the mean ± SD. Tissues where bacterial load was below the level of detection are given a value of “1” for visualization purposes.

To compare VC with infection by OG, we started with the method described by Barthel et al [14] for oral infection of streptomycin treated (hereafter referred to as strep+) B6 mice, a model that is now widely used. In addition to changing the inoculation method to VC, we made three other significant changes. First, since we were concerned that high levels of *Salmonella* might affect the palatability of chow, we used a low inoculum (10^4^) of *Salmonella*, although the dose most frequently used for oral infection is approximately 10^8^ [14, 27, 28]. Second, for mice infected by VC only, the streptomycin pretreatment was administered in the drinking water (final dilution of 5 mg/ml) for 24 h instead of by gavage (20 mg/mouse) 24 h prior to infection. B6 mice drink approximately 6 ml of water per 24 h [29], which results in an approximate total dose of 30 mg streptomycin. Third, whereas mice infected by OG were fasted for 4 h prior to each gavage (streptomycin pretreatment and infection) the mice infected by VC were fasted once for approximately 20 h before being given inoculated chow. A schematic comparing the two infection protocols is shown in Fig 2A. The experiment was designed to compare the two protocols (standard OG method vs VC method) rather than any individual step.

To infect mice by VC, single mice, which had been fasted for 20 h, were placed in a clean cage with no bedding and a fragment of *Salmonella*-inoculated chow was put on the floor of the cage. Chow was found, and consumed, more quickly if it was placed at the side of the cage due to the propensity of mice to run around the edge. After consuming the chow, mice were either returned to their original cage or transferred to a clean cage with bedding. For infection by OG, mice, which had been fasted for 4 h, were inoculated with *Salmonella* in SPGS (100 μl total vol) using a blunt gavage needle. All mice were euthanized at 3 days p.i. at which time they displayed very mild, if any, clinical signs of disease, although feces was frequently found on the walls of the cage, indicating wet stool. While all mice displayed similar colonization (Fig 2B), there were some differences, which may be due to the VC inoculation, the prolonged streptomycin treatment in the drinking water, the prolonged fasting period or any combination of these [30, 31]. Bacterial loads in tissues varied by 1–3 logs, with the exception of the feces and lungs of mice infected by OG which varied from below the limit of detection to 3.4×10^8^.and 8.7×10^3^ respectively. The presence of *Salmonella* in the lungs is not usually assessed but is not very surprising given the susceptibility of strep+ B6 mice to disseminated *Salmonella* infection. Strikingly, two mice infected by OG contained particularly high bacterial numbers in the lungs suggesting possible unintended tracheal delivery of bacteria [21]. Decreased systemic spread of *L*. *monocytogenes* following inoculation by contaminated food compared to OG, has been reported [24]. Similarly, decreased systemic spread was observed in mice infected by VC of water contaminated with *Salmonella* compared to OG [32]. Altogether, these three studies indicate that infection by VC is a viable alternative to OG.

### Inoculation of resistant mice by VC

**Fig 3:**
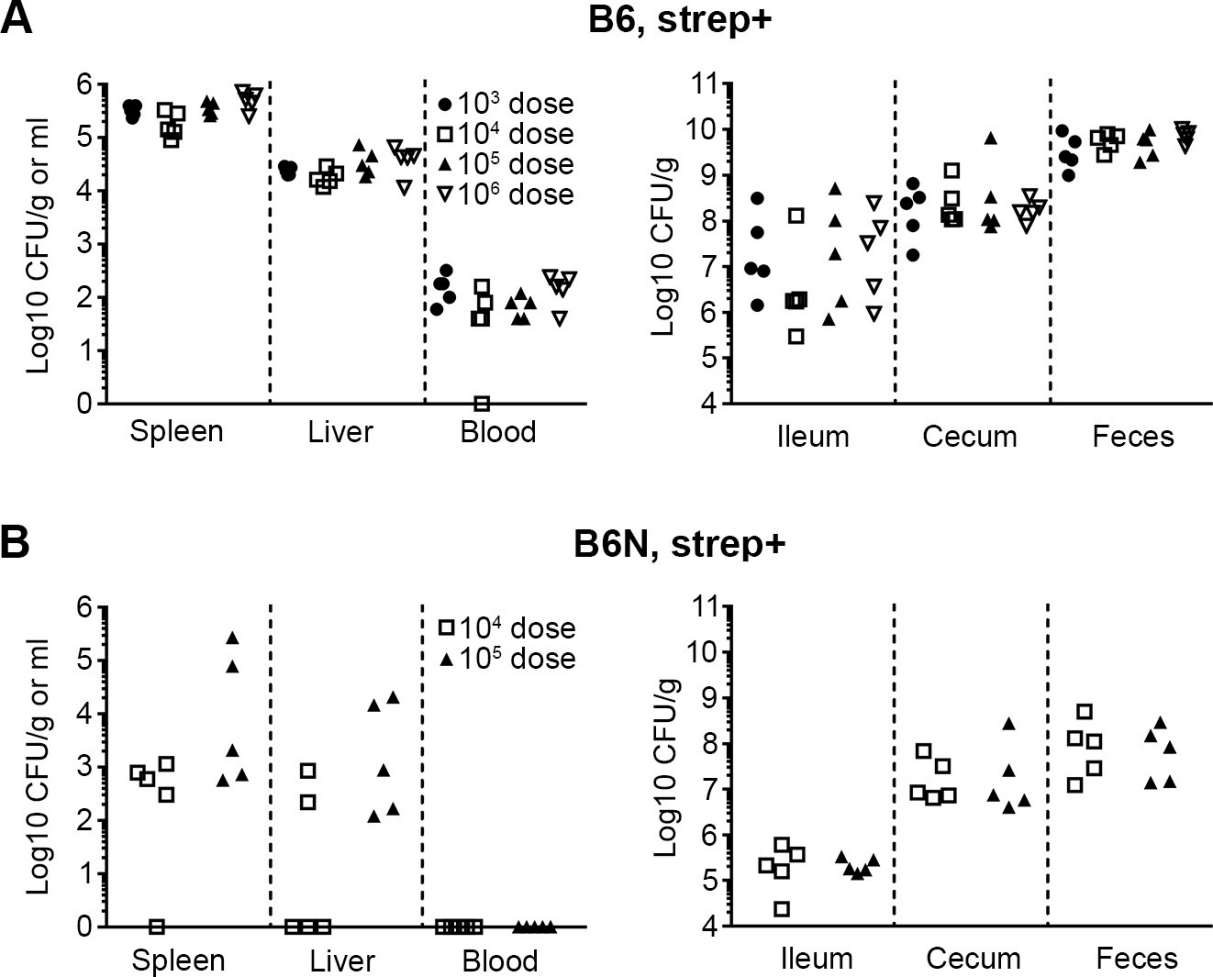
Colonization of B6 and B6N mice following VC inoculation. (A) Bacterial loads in tissues 3 days p.i. in strep+ B6 mice infected with indicated CFUs. (B) Bacterial loads in tissues 3 days p.i. in strep+ B6N mice infected with 10^4^ or 10^5^ CFUs. n = 5 mice and symbols represent individual mice. Tissues where bacterial load was below the level of detection are given a value of “1” for visualization purposes.

Transgenic B6N mice, which express a functional allele of Nramp1, or B6 mice not pretreated with antibiotic are more resistant to *Salmonella* than strep+ B6 mice, and higher inocula are used for infection by OG [6, 16]. Therefore, to test whether VC is compatible with higher inocula of *Salmonella* we first compared a dose range of 10^3^ to 10^6^ CFU in strep+ B6 mice. For doses of 10^5^ and 10^6^, the bacteria were rinsed once by centrifugation before dilution since we observed in preliminary experiments that otherwise mice were hesitant to consume chow inoculated with high doses (data not shown). By 3 days p.i. all mice were infected and the bacterial loads in organs revealed no dose dependence (Fig 3A), similar to what has been reported for mice given streptomycin in drinking water and infected by OG [33]. Bacterial loads were more variable in the gastrointestinal tract, particularly in the ileum (3×10^5^ to 5×10^8^ CFU/g), but there was no correlation with infectious dose.

In B6N mice a dose of 10^4^ resulted in variable colonization of systemic tissues, with CFUs below the limit of detection in the spleen and liver in 1 and 3 of 5 mice respectively, while 10^5^ resulted in consistent colonization, indicating that 10^5^ is the minimum dose required for 100% systemic colonization. As expected, the bacterial loads in general were lower in the B6N mice (Fig 3B), nonetheless, they followed the same trends seen in B6 mice, with the highest number of bacteria in the feces followed by the cecum, ileum, spleen, liver and blood similar to what has been shown previously [34].

**Fig 4:**
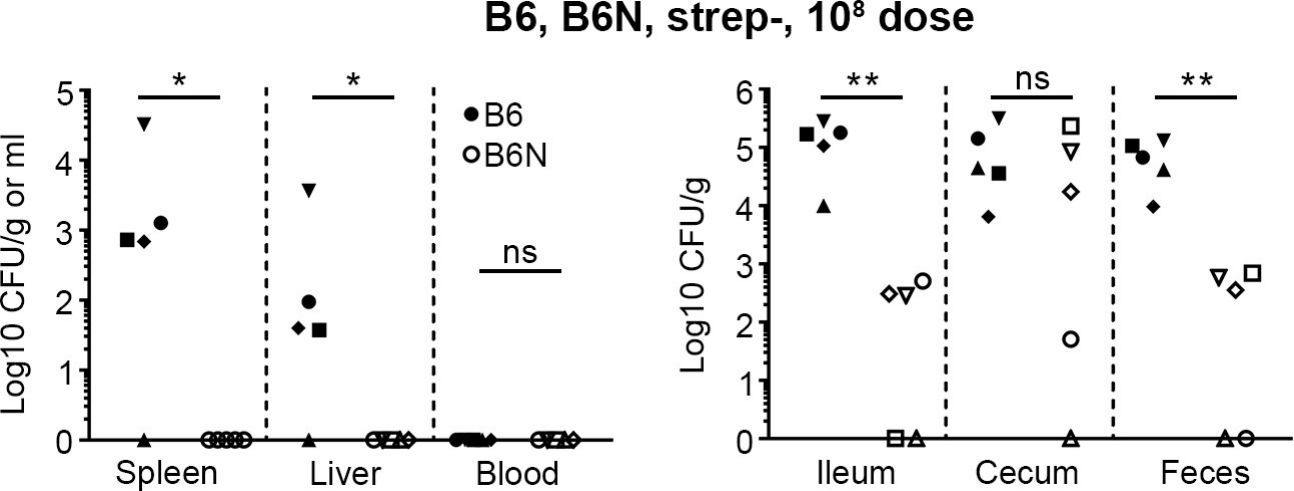
Infection of strep- mice. Bacterial numbers in tissues 3 days p.i. in strep- B6 and B6N mice infected with 10^8^ CFU *Salmonella*. n = 5 mice. Filled symbols represent individual B6 mice and open symbols represent individual B6N mice. Tissues where bacterial load was below the level of detection are given a value of “1” for visualization purposes. Asterix indicates statistical significance; * p < 0.05, ** p < 0.01, ns, not statistically different, two-tailed Mann-Whitney U test.

Mice with an intact microbiota are also much less susceptible to oral infection with *Salmonella*. Therefore, we also infected B6 and B6N mice that were not pretreated with streptomycin (hereafter referred to as strep-) by VC. For these experiments mice were infected with 10^8^ bacteria, the standard dose used in oral infections of mice with *Salmonella* (see e.g. [14, 28, 35]), using rinsed bacteria as in the previous experiment. At 3 days p.i. the intestinal tract of both B6 and B6N mice was colonized although B6N mice had lower bacterial loads (p < 0.05 for ileum and feces) apart from the cecum (Fig 4). In systemic tissues, no bacteria were detected in B6N mice and bacterial numbers were variable in B6 mice, with bacteria detectable in four out of five mice.

In summary, *Salmonella* infections of strep+ and strep- B6 and B6N mice by VC are in agreement with studies using OG. While it is difficult to determine the dose of nontyphoidal *Salmonella* required to cause gastroenteritis in humans, the dose is generally considered to be approximately 10^5^ to 10^6^ CFUs, although lower doses in susceptible individuals have been reported [36, 37]. Here we achieved colonization of strep+ mice using only 10^3^ organisms, indicating that this foodborne infection model can be used to investigate a range of relevant infection doses.

### Disease progression in mice infected by VC

**Fig 5:**
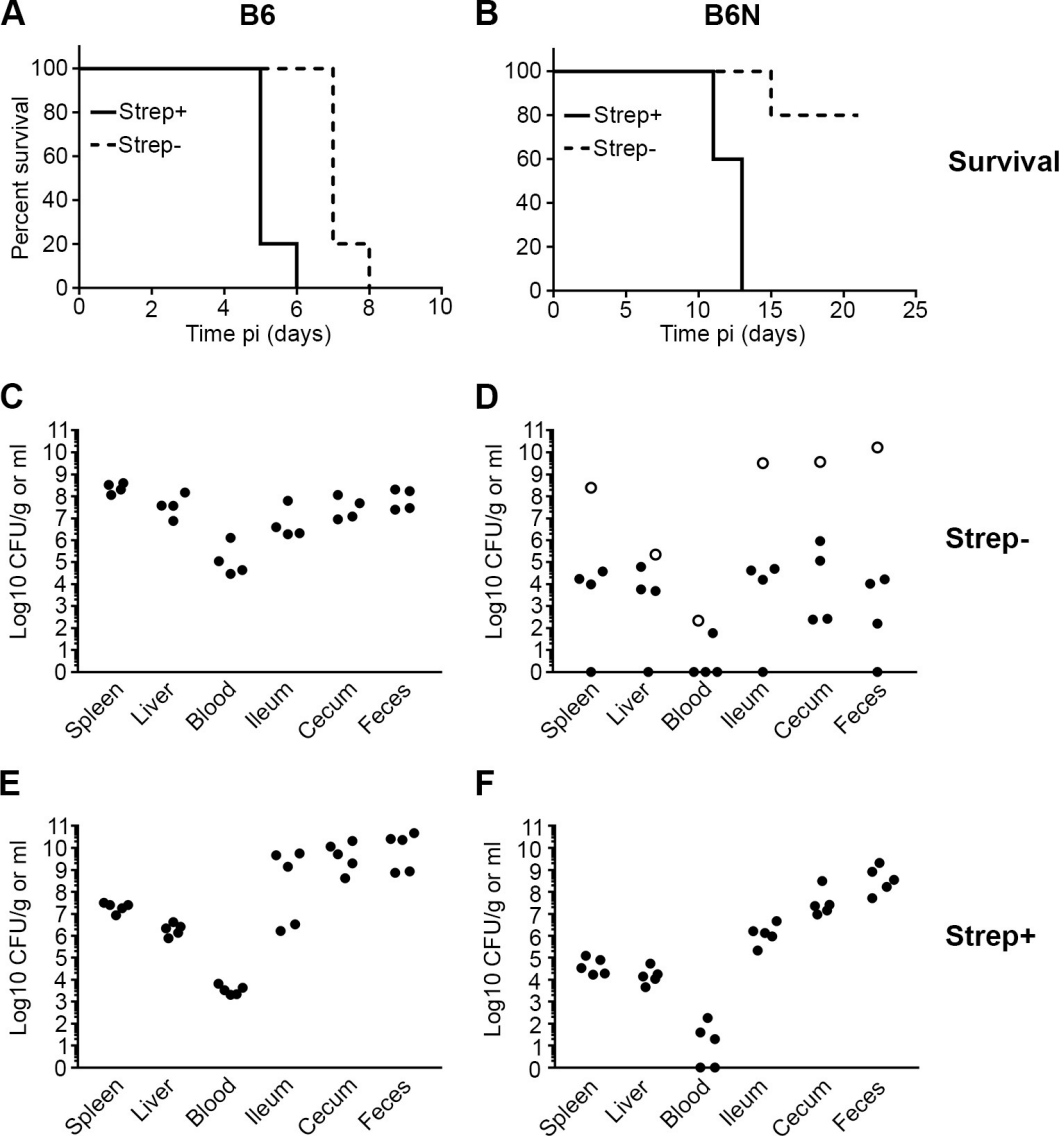
Kinetics of disease progression in mice inoculated by VC. Survival and bacterial loads of B6 (A, C, E) and B6N (B, D, F) mice. Strep- (not pretreated with antibiotic) and strep+ (pretreated with antibiotic in drinking water) mice were infected with 10^8^ or 10^4^ CFU respectively. n = 5 mice in each experiment and symbols represent individual mice. Open symbols in Fig 5D indicate the B6N mouse that was euthanized 15 days p.i. Tissues where bacterial load was below the level of detection are given a value of “1” for visualization purposes.

We next compared the disease progression in B6 and B6N mice infected by VC. Previously we showed that all B6 mice, infected via OG with no streptomycin pretreatment, developed clinical disease within 10 days (10^8^ CFU) compared to less than 30% of B6N by day 21 p.i. [16]. Here we obtained similar results with B6 and B6N mice infected by VC (Fig 5A and 5B). All B6 mice developed clinical signs and were euthanized on days 7 (4/5) and 8 (1/5) whereas only 1 out of 5 B6N mice developed clinical disease (day 15) and the rest (4/5) were clinically healthy when euthanized at day 21. At the time of euthanasia, all of the B6 were infected, while in B6N mice bacterial loads were sometimes below the limit of detection (Fig 5C and 5D). In contrast, when mice were pretreated with streptomycin and inoculated with 10^4^ CFU, both B6 and B6N mice developed clinical disease although this was slower in B6N (day 11–13 compared to day 5–6) (Fig 5A and 5B). All of these mice had systemic disease with detectable CFUs in the spleen and liver as well as in the intestine (Fig 5E and 5F). These data, together with the results of the early colonization studies (Figs 3 and 4), show that inoculation by VC does not alter the course of infection compared to inoculation by OG [6, 35, 38].

### Impact of fasting duration on foodborne infection

**Fig 6:**
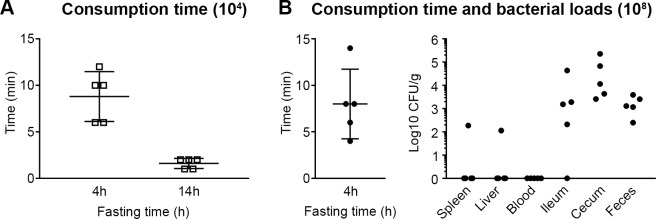
Fasting duration affects consumption time and bacterial loads. (A) Time required for mice to completely consume fragments of chow with the indicated inoculum and fasting time. Symbols represent individual mice and error bars represent the mean ± SD. 5 mice per experiment. (B) Consumption time (left panel) and bacterial loads (right panel) in tissues 3 days p.i. following 4 h of fasting and an inoculum of 10^8^ CFU *Salmonella*. n = 5 mice, symbols represent individual mice, error bars represent the mean ± SD.

Mice are routinely fasted before oral inoculation with *Salmonella*, although the fasting times vary from 2–16 h [6, 14, 16, 39, 40]. In their paper describing VC inoculation with *L*. *monocytogenes* Ghanem et al fasted mice for 16–24 h in cages with elevated wire flooring to prevent coprophagy [24, 26]. They also showed that 0–4 h of fasting was not sufficient to get bacterial colonization of the intestine but when food was withheld overnight (16 h) the colons of all mice were colonized [24]. Based on their findings we used a 20 h fast (initiated between 12-4pm) for the initial experiments, although mice were fasted in a clean cage with bedding instead of an elevated wire floor. While fasting overnight is a standard procedure, the nocturnal eating pattern of mice can lead to weight loss and stress [41]. Therefore, to determine whether a reduced fasting period could be used, without significantly affecting the time taken to consume inoculated chow, mice were fasted for 14 h (6pm– 8am) or 4 h (8am– 12pm) before inoculation (10^4^ CFUs). Mice fasted for 14 h consumed chow within 2 min, while those fasted for 4 h took up to 12 min (Fig 6A). Mice eat more in dark phase vs light phase so the short consumption time after 14 h may, at least in part, be due to fasting taking place overnight rather than during the day. However, since animal facilities generally discourage “after hours” access, unless necessary for animal welfare, we were unable to compare the effect of shorter night time fasting. To minimize the time taken to eat chow after 4 h fasting we slightly modified the feeding procedure. Mice were moved into individual clean cages, given a fragment of chow and then left undisturbed until consuming the whole chow fragment.

To determine whether mice would eat a higher inoculum after a short period of fasting, B6 mice were fasted for 4 h, and given chow inoculated with 10^8^ CFUs. Despite the higher inoculum, these mice consumed the fragments of chow in a time frame similar to mice fed 10^4^ CFUs (Fig 6B, left panel). Comparison of the bacterial loads in tissues from mice fasted for 4 h (Fig 6B, right panel) with mice fasted for 20 h (Fig 4) revealed less systemic dissemination at 3 days p.i. Since these experiments were not performed side by side the differences in organ loads may be due to experimental variation but overall these data indicate that shorter fasting times result in delayed, or less efficient, dissemination of *Salmonella*. In the method described for VC inoculation of mice with *L*. *monocytogenes* the mice sometimes had to be left undisturbed for up to 2 h to eat the offered food, even after 24 h of fasting [26]. In contrast, the mice in our study showed no reluctance to eat inoculated food, possibly because we used their regular chow rather than food they were unfamiliar with (buttered bread).

In summary, oral infection of mice by VC mimics the natural route of infection for *Salmonella* and results in reproducible colonization of tissues. This method is straightforward to carry out and may avoid the stress and potential adverse side effects of OG. Further refinements to the method are possible, such as; adjusting fasting times; the concentration of streptomycin in drinking water; and the time allowed for mice to access water containing streptomycin. This approach should also work for other intestinal pathogens.

## Materials and methods

### Ethics statement

All animal studies were carried out following the recommendations in the Guide for the Care and Use of Laboratory Animals, 8^th^ Edition (National Research Council), and were approved by the Rocky Mountain Laboratories Animal Care and Use Committee. Protocol number 2017-021-E. Animals were euthanized either before the development of clinical disease or at the defined humane endpoint (development of clinical disease: ruffled fur, hunched posture, lethargy).

### Bacterial strains and growth conditions

*Salmonella* Typhimurium strain SL1344 was used for all experiments. For infections, bacteria were grown in a 125 ml Erlenmeyer flask in 10 ml LB-Miller containing 100 μg/ml streptomycin for 18 h at 37°C, with shaking at 225 RPM. Bacteria were diluted in sterile SPGS to get the correct inoculum in 10 μl (e.g. to get 10^4^ CFU an overnight culture was diluted 1:5000). For inoculation of 10^5^ CFU or higher, a wash step was included prior to dilution. 1 ml of the overnight culture was centrifuged at 8000 X G for 2 min, and the bacterial pellet resuspended in 1 ml SPGS (10^5^−10^7^) or 0.5 ml (10^8^).

### Preparation of chow fragments for infection

Mouse chow pellets (2016 Teklad Global 16% Protein Rodent Diet, Envigo, Madison, Wisconsin USA), were broken into smaller fragments of about 4–5 mm in diameter and 30–45 mg by gentle tapping with a small hammer followed by trimming with forceps. Selected fragments were gently tested for physical integrity, by dropping from a height of 4–5 inches, before 10 μl of inoculum was pipetted onto the surface. Prepared pieces were kept separated in a petri dish during transport to the animal facility. One fragment of inoculated chow was retained for estimation of the dose by plating.

### Mouse infection by VC

The B6 mice used in this study were either from a colony of mice originally purchased from The Jackson Laboratory (Bar Harbor, ME) and maintained at the Rocky Mountain Laboratories or purchased from The Jackson Laboratory and used immediately after arrival. The B6N mice were from a colony maintained at the Rocky Mountain Laboratories [9]. Except where specified, mice had unlimited access to food and water. For streptomycin pretreatment the antibiotic (5 mg/ml) was added to drinking water 42–46 h prior to infection for 24 h. Mice were then moved to a clean cage (to limit coprophagy and access to cached food), containing normal drinking water but no chow. After a period of 18–22 h (typically 20 h) individual mice were put in a clean empty cage (without bedding material) and a fragment of inoculated chow placed on the floor of the cage next to a side. Typically, mice ate the fragment of chow immediately or within a couple of min. For the 4 h fasting mice were left undisturbed until the chow was eaten. Immediately after the inoculated chow was consumed, mice were returned to their cage with unlimited access to food and water.

### Mouse oral gavage infections

Mice were streptomycin treated 24 h before infection, using a blunt end straight size 20 gavage needle with 100 μl SPGS containing 200 mg/ml streptomycin. For *Salmonella* infection, mice were gavaged with bacteria in 100 μl SPGS. Mice were fasted for 4 h prior to all gavages. For infections without streptomycin treatment, mice were only fasted prior to feeding.

### Tissue collection and processing

Mice were euthanized by isoflurane inhalation followed by exsanguination. Tissues were collected in screwcap tubes containing 500 μl SPGS and 3–4 2.0 mm zirconia beads (BioSpec Products) and homogenized using a Bead Mill 24 (Fisher Scientific, 4.85 m/s for 20 seconds). Tubes were weighed before and after organ collection. CFUs were estimated by 10 μl spot plating of 10-fold dilutions on LB agar plates containing the appropriate antibiotic.
